# Supplementary material for: Identification, Classification, and Expression Analysis of the Triacylglycerol Lipase (TGL) Gene Family Related to Abiotic Stresses in Tomato
Source: Int J Mol Sci. 2021 Jan 30;22(3):1387. doi: 10.3390/ijms22031387 (PMC7866549; doi:10.3390/ijms22031387)
Supplement: Supplementary file 1 [file ijms-22-01387-s001.pdf]

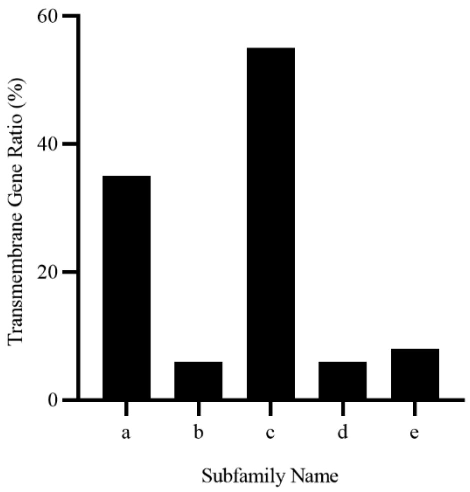

Figure S1. The transmembrane gene ratio of *SlTGL* gene subfamilies. Tansmembrane gene prediction were performed by using TMHMM Server v. 2.0 (https://services.healthtech.dtu.dk/.). All genes in each subfamily were involved in the prediction. a, b, c, d, and e represented subfamily a, subfamily b, subfamily c, subfamily d, and subfamily e.

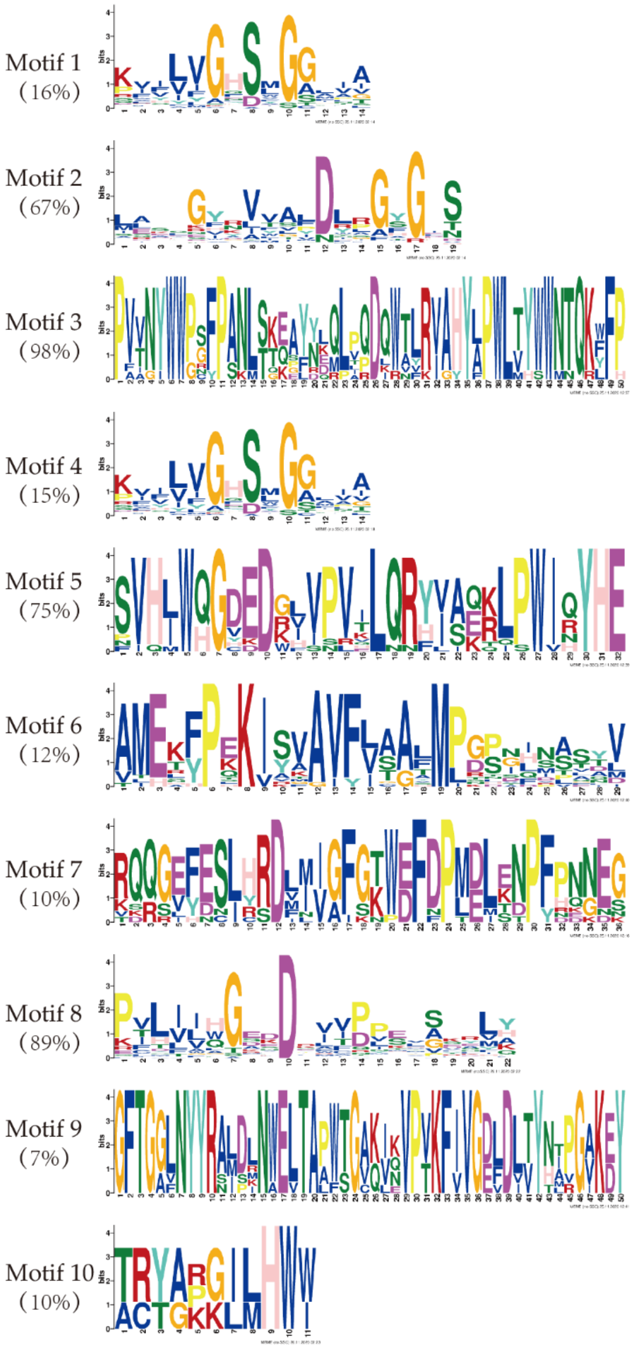

Figure S2. The consensus sequences of Motif 1-10 in SlTGL proteins. All motifs were scanned from 129 SlTGL amino acid sequences in MEME (http://meme-suite.org/tools/meme). Motif width between 10 to 50. The frequency of a motif occurrence per sequence is zero or one. The percentage under each motif represents the proportion of this motif in the TGL family.

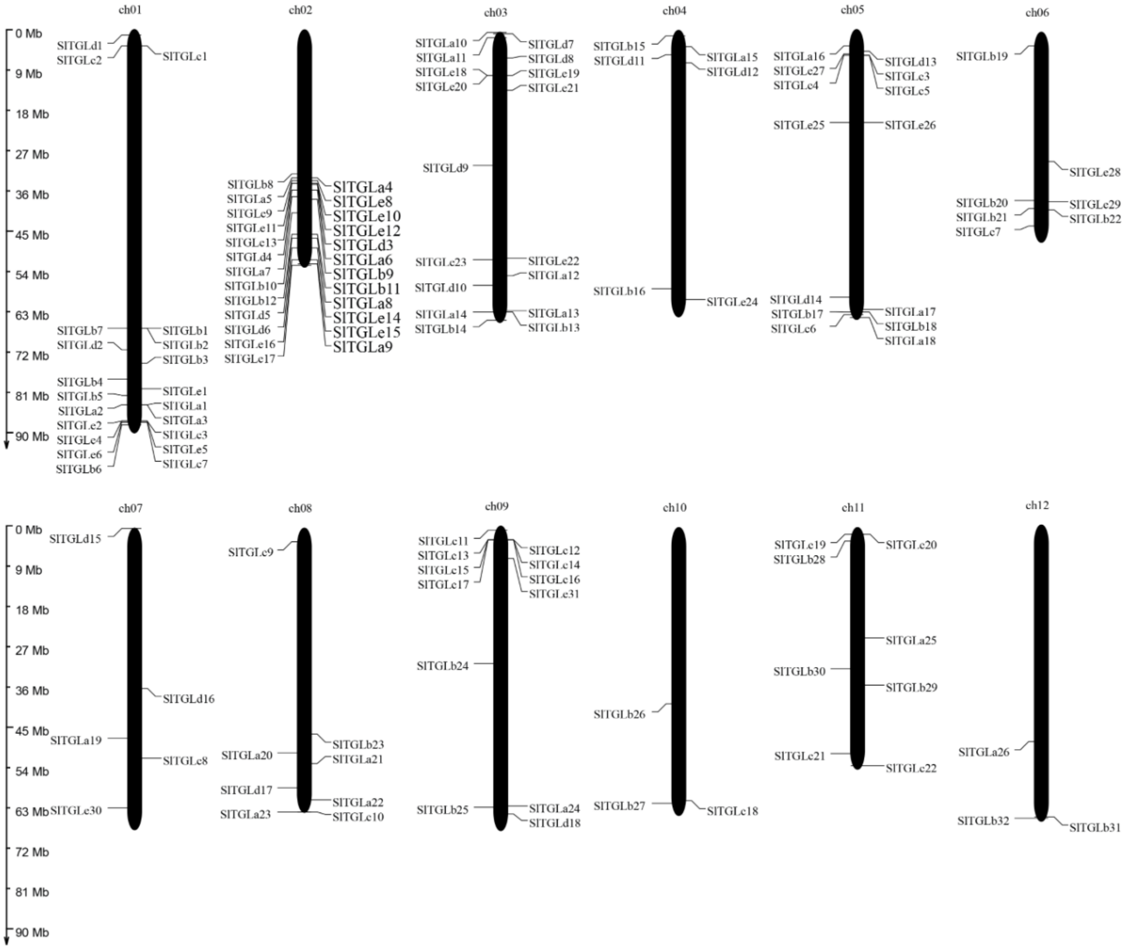

Figure S3. Chromosomal locations of *SlTGL* genes in *Solanum lycopersicum*. Chromosomal mapping was based on the physical position (Mb) in 12 tomato chromosomes. The chromosome number is indicated at the top of each bar. The positions of the tomato *SlTGL* genes in the chromosomes were obtained from Sol Genomics Network database (SGN, https://www.sgn.cornell.edu).

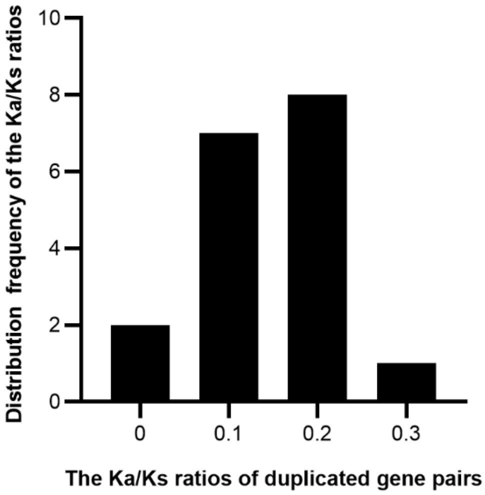

Figure S4. Histogram of distribution frequency of pairwise Ka/Ks ratios for homologous genes. The Ka/Ks ratios were calculated by TBtools (https://github.com/CJ-Chen/TBtools). The duplicated gene pairs were identified by MCscan.

Table S1. The detailed information of SlTGL members.

Gene	Sub-family	Annotated CDS	Genomic position	Chr	CDS	AA	pIs	MW	Subcellular localization
SlTGLa1	a	Solyc01g103650.3	92197324-92203584	1	1623	442	8.43	49.45	cyto: 6, E.R.: 3, plas: 2, chlo: 1, nucl: 1, mito: 1
SlTGLa2	a	Solyc01g103660.4	92209732-92216532	1	1838	551	8.31	61.81	E.R.: 4, nucl: 3, cyto: 3, mito: 1, plas: 1, vacu: 1, pero: 1
SlTGLa3	a	Solyc01g103670.4	92217828-92226403	1	1946	532	5.86	59.72	plas: 8, E.R.: 3, nucl: 1, extr: 1, vacu: 1
SlTGLa4	a	Solyc02g063160.4	35233966-35239703	2	1397	382	6.91	43.16	cyto: 12, nucl: 1, mito: 1
SlTGLa5	a	Solyc02g063200.3	35246162-35252359	2	1044	347	8.5	38.32	cyto: 6, nucl: 3, cysk: 2, vacu: 1.5, E.R._vacu: 1.5, plas: 1
SlTGLa6	a	Solyc02g068070.4	38118498-38123349	2	1544	418	5.66	46.12	chlo: 5, cyto: 3, E.R.: 2, nucl: 1, mito: 1, extr: 1, vacu: 1
SlTGLa7	a	Solyc02g069930.1	39709565-39708606	2	960	319	8.4	36.05	pero: 9, cyto_nucl: 3, nucl: 2.5, cyto: 2.5
SlTGLa8	a	Solyc02g086040.2	48839772-48842414	2	849	282	6.71	31.73	cyto: 5, nucl: 4, cysk: 3, extr: 1, E.R._vacu: 1
SlTGLa9	a	Solyc02g094040.3	54686908-54690119	2	1374	403	6.96	44.74	nucl: 3, E.R.: 3, chlo: 2, cyto: 2, vacu: 2, mito: 1, plas: 1
SlTGLa10	a	Solyc03g005020.3	11260524-1127010	3	1264	421	7.43	39.42	
SlTGLa11	a	Solyc03g006560.3	1128702-1130011	3	1241	313	8.08	35.42	cyto: 9, nucl: 4, cysk: 1
SlTGLa12	a	Solyc03g098020.4	60369018-60376150	3	1829	470	9.58	52.48	chlo: 10, mito: 3, golg: 1
SlTGLa13	a	Solyc03g119720.4	68262870-68269514	3	2653	768	9.11	87.29	plas: 6, vacu: 3, nucl: 1, cyto: 1, mito: 1, E.R.: 1, pero: 1
SlTGLa14	a	Solyc03g119980.3	68494252-68491760	3	1386	327	5.91	36.29	nucl: 7, mito: 3, cyto: 2, plas: 1, cysk: 1
SlTGLa15	a	Solyc04g010250.3	3577656-3583500	4	1979	319	6.38	36.58	cyto: 8, nucl: 3, cysk: 3
SlTGLa16	a	Solyc05g009390.3	3548741-3544526	5	1454	397	8.82	44.64	chlo: 11, cyto: 2, mito: 1
SlTGLa17	a	Solyc05g053750.3	63774196-63780154	5	1197	398	5.2	44.6	chlo: 6, extr: 4, E.R.: 2, plas: 1, pero: 1
SlTGLa18	a	Solyc05g05600.3	65396461-65398412	5	1456	335	6.23	37.16	cyto: 9, nucl: 2, cysk: 2, golg: 1
SlTGLa19	a	Solyc07g039510.4	47624851-47617843	7	1212	259	5.76	29	cyto: 10, chlo: 1, nucl: 1, mito: 1, E.R.: 1
SlTGLa20	a	Solyc08g063070.3	52625247-52632402	8	1253	288	8.18	32.45	pero: 11, cyto: 3
SlTGLa21	a	Solyc08g066440.3	6287641-62876824	8	1257	275	7.53	23.57	
SlTGLa22	a	Solyc08g079450.3	62967637-62975950	8	1541	405	5.72	44.84	chlo: 4, nucl: 4, cyto: 2, plas: 2, E.R.: 2
SlTGLa23	a	Solyc08g083140.4	65681856-65688865	8	1480	418	7.03	46.51	chlo: 6, nucl: 6, cyto: 2
SlTGLa24	a	Solyc09g075140.3	25766015-25796500	9					
SlTGLa25	a	Solyc11g032200.2	25797006-25817118	11	1206	401	8.82	45.4	chlo: 7, extr: 3, vacu: 2, cyto: 1, plas: 1
SlTGLa26	a	Solyc12g038160.2	48802448-48795765	12	2073	690	6.46	77.71	plas: 10.5, cyto_plas: 6, E.R.: 2, mito: 1
SlTGLb1	b	Solyc01g066450.3	74421456-74424175	1	1215	335	6.01	38.08	cyto: 14
SlTGLb2	b	Solyc01g066457.1	744225871-74427076	1	1205	325	5.84	36.92	cyto: 11, nucl: 1, E.R.: 1, golg: 1
SlTGLb3	b	Solyc01g088090.3	82931116-82927775	1	1919	512	7	57.63	chlo: 12, nucl: 1, cysk: 1
SlTGLb4	b	Solyc01g095120.3	86506984-86512818	1	1638	514	7.19	57.27	chlo: 8, mito: 6
SlTGLb5	b	Solyc01g099910.4	90032830-90029882	1	1170	325	5.84	36.92	pero: 7, E.R.: 3, plas: 2, chlo: 1, vacu: 1
SlTGLb6	b	Solyc01g109720.3	96606949-96604848	1	1317	295	8.33	33.26	cyto: 9.5, cyto_E.R.: 5.5, nucl: 2, cysk: 2
SlTGLb7	b	Solyc01g150144.1	90125840-90127046	1	1206	325	5.89	36.93	cyto: 11, nucl: 1, extr: 1, E.R.: 1

SlTGLb8	b	Solyc02g062610.3	34331402-34326437	2	1526	372	6.2	41.19	chlo: 9, cyto: 4, nucl: 1
SlTGLb9	b	Solyc02g070490.4	40226768-40224187	2	1446	387	6.65	42.39	chlo: 5, mito: 3, vacu: 2, nucl: 1, cyto: 1, plas: 1, extr: 1
SlTGLb10	b	Solyc02g078570.3	43236921-43234612	2	1480	315	5.36	35.62	cyto: 7, chlo: 2, nucl: 2, E.R.: 1, cysk: 1, golg: 1
SlTGLb11	b	Solyc02g085070.1	48083454-48085548	2	1092	363	8.32	40.86	chlo: 11.5, chlo_mito: 7, mito: 1.5, nucl: 1
SlTGLb12	b	Solyc02g085180.5	48161687-48158880	2	1145	332	8.87	36.8	cyto: 8, chlo: 2, nucl: 2, mito: 1, cysk: 1
SlTGLb13	b	Solyc03g119990.4	68503372-68508586	3	2252	633	9.16	69.01	plas: 11, E.R.: 2, vacu: 1
SlTGLb14	b	Solyc03g123390.4	70260512-70264701	3	1337	370	9.03	43	cyto: 6, mito: 2.5, nucl: 2, cyto_mito: 2, plas: 1.5, golg_plas: 1.5, cysk: 1
SlTGLb15	b	Solyc04g007450.4	1119419-1113432	4	1820	612	5.64	60.8	chlo: 9, nucl: 2, mito: 2, cysk: 1
SlTGLb16	b	Solyc04g074220.4	60214975-60219706	4	1176	248	5.8	28.11	cyto: 7, chlo: 3, nucl: 2, plas: 2
SlTGLb17	b	Solyc05g054315.1	63640191- 63645607	5	966	321	5.6	36.4	cyto: 10, chlo: 1, nucl: 1, extr: 1, cysk: 1
SlTGLb18	b	Solyc05g054350.3	64278493-64275459	5	1225	321	5.77	36.22	cyto: 6, E.R.: 5, mito: 2, pero: 1
SlTGLb19	b	Solyc06g009170.3	3108490-3113302	6	1354	318	6.06	36.21	cyto: 7, extr: 2, E.R.: 2, chlo: 1, mito: 1, vacu: 1
SlTGLb20	b	Solyc06g064640.3	40278200-40292413	6	1679	378	8.99	41.76	chlo: 8, mito: 5, plas: 1
SlTGLb21	b	Solyc06g067890.3	42079659-42085923	6	2470	636	8.72	69.58	plas: 10, E.R.: 3, golg: 1
SlTGLb22	b	Solyc06g068220.3	42261088-42267698	6	1553	394	6.2	43.6	chlo: 12, nucl: 1, mito: 1
SlTGLb23	b	Solyc08g061240.3	48255185-48254517	8	669	222	9.06	25	nucl: 11, chlo: 2, mito: 1
SlTGLb24	b	Solyc09g047900.2	33689158-33695213	9	1155	384	6.16	42.66	chlo: 11.5, chlo_mito: 7, mito: 1.5, nucl: 1
SlTGLb25	b	Solyc09g075300.3	67029977-67033875	9	1185	318	5.62	36.4	cyto: 8, mito: 2, nucl: 1, E.R.: 1, cysk: 1, golg: 1
SlTGLb26	b	Solyc10g047340.2	40581634-40582766	10	900	299	6.66	34.32	mito: 7.5, cyto_mito: 4.5, chlo: 4, nucl: 2
SlTGLb27	b	Solyc10g083270.3	63101923-63105022	10	996	331	7.17	36.98	mito: 7.5, chlo_mito: 6, chlo: 3.5, nucl: 2, cyto: 1
SlTGLb28	b	Solyc11g009000.2	3151023-3152011	11	678	225	8.77	25.54	chlo: 12, mito: 2
SlTGLb29	b	Solyc11g042430.2	36614861-36623795	11	1182	393	8.18	43.39	chlo: 5, mito: 4, pero: 3, nucl: 2
SlTGLb30	b	Solyc11g044310.2	33045449-33034623	11	1191	400	6.2	45.14	chlo: 7.5, chlo_mito: 7.5, mito: 6.5
SlTGLb31	b	Solyc12g098660.3	66091429-66095890	12	1122	373	7.48	41.59	chlo: 6, nucl: 3, cyto: 2, plas: 1, extr: 1, golg: 1
SlTGLb32	b	Solyc12g099430.2	66584035-66587327	12	972	323	6.81	36.79	cyto: 11, chlo: 1, extr: 1, cysk: 1
SlTGLc1	c	Solyc01g009480.4	3706348-3702854	1	1332	324	8.7	37.39	cyto: 8, nucl: 2, chlo: 1, mito: 1, extr: 1, cysk: 1
SlTGLc2	c	Solyc01g009500.4	3739772-3743748	1	1587	354	7.03	40.53	chlo: 4, mito: 3.5, cyto_mito: 2.5, nucl: 2, extr: 2, cyto: 1, plas: 1
SlTGLc3	c	Solyc05g012350.4	5613049-5616480	5	1360	255	8.47	28.31	cyto: 5, chlo: 3, extr: 2, cysk: 2, nucl: 1, mito: 1
SlTGLc4	c	Solyc05g012360.3	5622420-5627115	5	1311	362	8.38	40.14	chlo: 8, cyto: 3, nucl: 1, plas: 1, cysk: 1
SlTGLc5	c	Solyc05g012370.3	5634334-5638589	5	1451	351	6.71	38.77	chlo: 12.5, chlo_mito: 7, plas: 1
SlTGLc6	c	Solyc05g055290.3	64990554-64987424	5	1231	347	8.46	39.85	chlo: 5, vacu: 3, golg: 2, nucl: 1, mito: 1, plas: 1, extr: 1
SlTGLc7	c	Solyc06g074330.3	45955461-45953145	6	1466	354	6.51	40.16	cyto: 9, nucl: 2, chlo: 1, plas: 1, cysk: 1
SlTGLc8	c	Solyc07g041200.3	51988393-51982077	7	1332	325	8.32	36.3	chlo: 3, extr: 3, mito: 2, vacu: 2, nucl: 1, cyto: 1, E.R.: 1, golg: 1
SlTGLc9	c	Solyc08g008610.3	3008230-3003194	8	1704	470	8.91	53.63	plas: 8, E.R.: 3, extr: 1, vacu: 1, golg: 1
SlTGLc10	c	Solyc08g083190.3	65726318-65723499	8	1653	449	8.75	50.57	plas: 5, E.R.: 5, chlo: 1, extr: 1, vacu: 1, pero: 1
SlTGLc11	c	Solyc09g007380.3	972929-976824	9	1913	526	5.55	59.35	chlo: 4, nucl: 2.5, plas: 2, vacu: 2, cysk_nucl: 2, cyto: 1, mito: 1, E.R.: 1
SlTGLc12	c	Solyc09g009500.3	2924458-2918891	9	1463	370	9.07	41.63	chlo: 6, mito: 3, golg: 2, nucl: 1, cyto: 1, plas: 1

SlTGLc13	c	Solyc09g009510.4	2929510-2924885	9	1461	409	8.48	46.56	cyto: 4, nucl: 2, mito: 2, E.R.: 2, plas: 1, extr: 1, vacu: 1, pero: 1
SlTGLc14	c	Solyc09g009520.3	2932883-2929991	9	1510	376	9.13	42.22	chlo: 4, mito: 4, golg: 2, nucl: 1, cyto: 1, plas: 1, pero: 1
SlTGLc15	c	Solyc09g009530.4	2939724-2936861	9	1543	348	5.95	39.88	chlo: 3, vacu: 3, E.R.: 2, nucl: 1, cyto: 1, mito: 1, plas: 1, extr: 1, golg: 1
SlTGLc16	c	Solyc09g009540.4	2953213-2948975	9	1372	366	6.81	41.35	nucl: 3, vacu: 3, chlo: 2, E.R.: 2, cyto: 1, plas: 1, extr: 1, golg: 1
SlTGLc17	c	Solyc09g009550.4	2963265-296089	9	1243	349	6.32	39.28	vacu: 4, chlo: 2, extr: 2, E.R.: 2, golg: 2, nucl: 1, cyto: 1
SlTGLc18	c	Solyc10g081450.2	62506953-62510182	10	1272	423	7.53	48.25	plas: 5, vacu: 3, E.R.: 3, cyto: 1, mito: 1, extr: 1
SlTGLc19	c	Solyc11g007030.3	1521058-1516044	11	1266	421	8.26	48.17	chlo: 8, vacu: 3, nucl: 1, E.R.: 1, pero: 1
SlTGLc20	c	Solyc11g007040.2	1525575-1521934	11	1218	405	9.09	45.98	chlo: 13, mito: 1
SlTGLc21	c	Solyc11g067260.3	52996336-53001292	11	1161	386	9.05	43.57	chlo: 11, mito: 2.5, cyto_mito: 2
SlTGLc22	c	Solyc11g072440.2	55618102-55614355	11	1626	541	6	60.77	chlo: 4, plas: 3, vacu: 2, E.R.: 2, nucl: 1, cyto: 1, mito: 1
SlTGLd1	d	Solyc01g006480.3	1074151-1078328	1	1119	236	6.65	25.53	cyto: 6, nucl: 4, extr: 3, cysk: 1
SlTGLd2	d	Solyc01g080270.3	79516162-79496502	1	2231	568	4.73	63.14	cyto: 7, chlo: 5, nucl: 2
SlTGLd3	d	Solyc02g065700.3	36856743-36853200	2	1711	281	5.4	31.15	chlo: 4, nucl: 3, cyto: 3, mito: 1, extr: 1, vacu: 1, cysk: 1
SlTGLd4	d	Solyc02g067920.4	38007199-38013034	2	1478	370	9.14	42.46	cyto: 8, nucl: 3, pero: 2, extr: 1
SlTGLd5	d	Solyc02g086220.3	48953869-48948084	2	1569	340	8.95	38.03	chlo: 4, cyto: 4, plas: 3, mito: 1, cysk: 1, golg: 1
SlTGLd6	d	Solyc02g089320.3	51178522-51182343	2	1306	359	8.58	40.23	chlo: 10, cyto: 2, extr: 1, vacu: 1
SlTGLd7	d	Solyc03g005340.4	1204060- 1196973	3	2758	829	6.67	90.76	chlo: 11, nucl: 1, plas: 1, pero: 1
SlTGLd8	d	Solyc03g034270.3	6015507-6008882	3	1422	340	9.31	37.8	cyto: 5.5, cyto_nucl: 5, nucl: 3.5, chlo: 3, mito: 1, plas: 1
SlTGLd9	d	Solyc03g063290.3	33959869-33958830	3	946	227	8.68	24.06	hlo: 4, cyto: 3.5, cyto_nucl: 3.5, nucl: 2.5, extr: 2, mito: 1, vacu: 1
SlTGLd10	d	Solyc03g111770.3	62404229-62399613	3	1462	371	9.16	42.77	cyto: 7, chlo: 5, plas: 1, pero: 1
SlTGLd11	d	Solyc04g015300.3	5508593-5497992	4	2110	490	6.54	54.66	chlo: 6, nucl: 4, mito: 2, cyto: 1, cysk: 1
SlTGLd12	d	Solyc04g016360.3	7158486-7165657	4	1247	284	5.85	32.09	cyto: 10, mito: 2, chlo: 1, extr: 1
SlTGLd13	d	Solyc05g010680.2	4900485-4897375	5	1152	383	6.16	43.65	nucl: 10, cyto: 2, chlo: 1, extr: 1
SlTGLd14	d	Solyc05g050710.3	60840680-60838060	5	1664	397	8.82	44.65	pero: 4, cyto: 3.5, cyto_nucl: 3.5, chlo: 3, nucl: 2.5, golg: 1
SlTGLd15	d	Solyc07g005130.3	145544-151215	7	1353	295	9.15	33.41	cyto: 9, plas: 2, chlo: 1, nucl: 1, cysk: 1
SlTGLd16	d	Solyc07g032170.3	36036253-36039504	7	842	168	9.2	19.01	cyto: 6.5, cyto_nucl: 5, mito: 4, nucl: 2.5, chlo: 1
SlTGLd17	d	Solyc08g076350.3	60317705-60310093	8	1583	390	8.72	43.33	chlo: 8, cyto: 4, nucl: 1, mito: 1
SlTGLd18	d	Solyc09g083130.3	68810472-68803861	9	2701	767	6.01	83.55	cyto: 6, mito: 3, nucl: 2, cysk: 2, chlo: 1
SlTGLe1	e	Solyc01g098110.4	88704352-88719638	1	2273	689	7.79	77.12	cyto: 8.5, cyto_E.R.: 5, chlo: 2, nucl: 1, pero: 1, golg: 1
SlTGLe2	e	Solyc01g108680.4	95922791-95924890	1	1191	366	6.85	40.6	pero: 10, nucl: 2, chlo: 1, cyto: 1
SlTGLe3	e	Solyc01g108740.3	95953723-95952490	1	1018	288	9.39	32.13	chlo: 11, cyto: 1, mito: 1, E.R.: 1
SlTGLe4	e	Solyc01g108750.2	95955876-95954713	1	967	297	6.87	32.81	plas: 4, vacu: 3, E.R.: 3, nucl: 2, cyto: 1, golg: 1
SlTGLe5	e	Solyc01g108780.4	95971618-95970449	1	994	265	6.39	29.03	chlo: 12, mito: 1, vacu: 1

SlTGLe6	e	Solyc01g108810.3	95992298-95989170	1	943	269	6.3	29.67	cyto: 5, mito: 3, chlo: 2, nucl: 1, pero: 1, cysk: 1, E.R._vacu: 1
SlTGLe7	e	Solyc01g108820.1	95994206-95993151	1	768	255	9.03	28.32	chlo: 8, vacu: 4.5, E.R._vacu: 3, cyto: 1
SlTGLe8	e	Solyc02g064760.3	35858378-35860385	2	702	117	6.15	13.06	chlo: 8, mito: 3, nucl: 1, cyto: 1, vacu: 1
SlTGLe9	e	Solyc02g064770.4	35880668-35885164	2	1188	295	5.23	33	cyto: 10, nucl: 2, chlo: 1, E.R.: 1
SlTGLe10	e	Solyc02g065240.3	36416349-36418203	2	1152	264	5.73	29.6	cyto: 8.5, cyto_E.R.: 5, chlo: 3, nucl: 1, extr: 1
SlTGLe11	e	Solyc02g065250.2	36422010-36422996	2	594	197	5.48	22.4	cyto: 6.5, cyto_E.R.: 4, chlo: 3, nucl: 1, mito: 1, extr: 1, cysk: 1
SlTGLe12	e	Solyc02g065260.4	36435241-36437123	2	750	170	4.96	19.15	nucl: 4, cyto: 3.5, cyto_E.R.: 2.5, chlo: 2, plas: 2, extr: 1, cysk: 1
SlTGLe13	e	Solyc02g065280.3	36443070-36448965	2	1036	265	5.56	30.19	cyto: 4, chlo: 3, nucl: 3, mito: 2, extr: 1, pero: 1
SlTGLe14	e	Solyc02g089060.3	50960828-50966231	2	1543	379	9.21	41.45	chlo: 8, mito: 3.5, cyto_mito: 2.5, nucl: 1, cysk: 1
SlTGLe15	e	Solyc02g092760.3	53721353-53722870	2	1109	275	4.94	30.49	cysk: 6, cyto: 3, nucl: 2, extr: 2, mito: 1
SlTGLe16	e	Solyc02g092770.3	53725871-53727869	2	1159	272	4.79	30.22	cyto: 6, nucl: 4, cysk: 2, chlo: 1, extr: 1
SlTGLe17	e	Solyc02g094430.4	54961901-54953503	2	2286	685	7.09	76.66	chlo: 12.5, chlo_mito: 7.5, mito: 1.5
SlTGLe18	e	Solyc03g044740.4	10460420-10465259	3	1113	301	6.92	34.73	nucl: 7, cyto: 3, plas: 1.5, golg_plas: 1.5, mito: 1, cysk: 1
SlTGLe19	e	Solyc03g044790.3	10543209-10540964	3	956	262	5.52	29.63	cyto: 6, nucl: 3, chlo: 1, mito: 1, extr: 1, vacu: 1, cysk: 1
SlTGLe20	e	Solyc03g044810.2	10567175-10566807	3	369	122	6.02	13.36	cyto: 11, chlo: 2, cysk: 1
SlTGLe21	e	Solyc03g070380.3	16206619-16211471	3	1028	264	5.64	29.93	cyto: 5.5, cyto_E.R.: 4, chlo: 2, nucl: 2, extr: 2, E.R.: 1.5, mito: 1
SlTGLe22	e	Solyc03g095290.3	56315780-56323128	3	1839	484	8.69	53.37	plas: 7, chlo: 2, vacu: 2, E.R.: 2, pero: 1
SlTGLe23	e	Solyc03g095550.2	56759541-56757347	3	822	273	6	30.59	cyto: 5, nucl: 3, golg: 3, chlo: 1, mito: 1, vacu: 1
SlTGLe24	e	Solyc04g077860.4	62744584-62742302	4	1178	304	9.68	34.23	chlo: 6, nucl: 3, mito: 3, cyto: 2
SlTGLe25	e	Solyc05g018403.1	20787617- 20789467	5	810	269	5.57	30.49	nucl: 10, cyto: 3, mito: 1
SlTGLe26	e	Solyc05g018413.1	20814474- 20819162	5	810	269	5.57	30.55	nucl: 10, cyto: 3, mito: 1
SlTGLe27	e	Solyc05g012180.3	5449535-5446238	5	1272	419	9.57	47.21	chlo: 5, mito: 5, nucl: 2.5, cyto_nucl: 2, golg_plas: 1
SlTGLe28	e	Solyc06g048570.3	31262771-31260220	6	1244	280	5.42	31.03	cyto: 5, golg: 3, vacu: 2, E.R._plas: 2, plas: 1.5, E.R.: 1.5, nucl: 1
SlTGLe29	e	Solyc06g064870.4	40437593-40442243	6	1527	350	8.22	39.13	cyto: 9, nucl: 4, extr: 1
SlTGLe30	e	Solyc07g054880.4	63059126-63060350	7	944	283	5.68	31.45	chlo: 6, cyto: 3, extr: 3, vacu: 2
SlTGLe31	e	Solyc09g014970.4	7327860-7326642	9	1053	294	9.27	32.87	chlo: 5, nucl: 2, cyto: 2, E.R.: 2, mito: 1, plas: 1, extr: 1

Note: Chr, chromosome; CDS, length of coding sequence; AA, number of amino acid; pIs, theoretical isoelectric point; MW, molecular weight, KDa; The subcellular location of tomato lipase proteins was predicted using WoLF PSORT (http://www.genscript.com/psort/wolf_psort.html). Nucl, nucleus; Mito, mitochondria; Chlo, chloroplast; Cyto, cytosol; E.R, endoplasmic reticulum; Cysk, cytoskeleton; Plas, plasma membrane; Vacu: Tonoplast; Extr, extracellular. The numbers under the column heading 'Subcellular localization' reflect probability of potential subcellular loclizations with the Testk used for kNN set at 14.

Table S2. Ka/Ks analysis for the duplicated *SlTGL* gene pairs.

Duplicated gene 1	Duplicated gene 2	Ka	Ks	Ka/Ks	Purifying selection	Duplicate type
SlLIPc11	*SlLIPc22*	0.380	1.793	0.212	Yes	Segmental
SlLIPc9	*SlLIPc10*	0.145	0.905	0.160	Yes	Segmental
SlLIPa19	*SlLIPa20*	0.280	2.867	0.098	Yes	Segmental
SlLIPb19	*SlLIPb25*	0.216	1.600	0.135	Yes	Segmental
SlLIPc6	*SlLIPc19*	0.316	1.375	0.230	Yes	Segmental
SlLIPb16	*SlLIPb28*	0.330	5.664	0.058	Yes	Segmental
SlLIPb13	*SlLIPb21*	0.152	0.495	0.307	Yes	Segmental
SlLIPa13	*SlLIPa26*	0.127	0.620	0.204	Yes	Segmental
SlLIPe23	*SlLIPe28*	0.181	0.899	0.201	Yes	Segmental
SlLIPa9	*SlLIPa10*	0.218	0.883	0.246	Yes	Segmental
SlLIPe14	*SlLIPe27*	0.366	2.167	0.169	Yes	Segmental
SlLIPd5	*SlLIPd8*	0.101	0.764	0.132	Yes	Segmental
SlLIPd3	*SlLIPd6*	0.092	0.569	0.162	Yes	Segmental
SlLIPe8	*SlLIPe15*	0.214	0.943	0.227	Yes	Segmental
SlLIPb8	*SlLIPb11*	0.171	0.614	0.279	Yes	Segmental
SlLIPb5	*SlLIPb25*	0.320	1.568	0.204	Yes	Segmental
SlLIPb1	*SlLIPb17*	0.306	1.603	0.191	Yes	Segmental
SlLIPb1	*SlLIPb32*	0.214	1.339	0.160	Yes	Segmental

Table S3. The stress response-related *cis*-acting elements in putative promoter regions of *SlTGL* genes.

cis-acting elements	Number of genes	Function of the cis-acting element	Type of *cis*-elements
as-1	*SlLIPa26; SlLIPc21; SlLIPc19; SlLIPb25; SlLIPb24; SlLIPc15; SlLIPc14; SlLIPc13; SlLIPa23; SlLIPa22; SlLIPd17; SlLIPa21; SlLIPc9; SlLIPd16; SlLIPb22; SlLIPd13; SlLIPe26; SlLIPc5; SlLIPc4; SlLIPc3; SlLIPe27; SlLIPe24; SlLIPd11; SlLIPa14; SlLIPa13; SlLIPa12; SlLIPe22; SlLIPe21; SlLIPe19; SlLIPd8; SlLIPd7; SlLIPe17; SlLIPa9; SlLIPe16; SlLIPd5; SlLIPa8; SlLIPb12; SlLIPd3; SlLIPb8; SlLIPe4; SlLIPe3; SlLIPa3; SlLIPa1; SlLIPe1; SlLIPd2;*	N/A	Plant growth and development
WRE3	*SlLIPc22; SlLIPa25; SlLIPc16; SlLIPc15; SlLIPc12; SlLIPd17; SlLIPe30; SlLIPa17; SlLIPb13; SlLIPe21; SlLIPd7; SlLIPe17; SlLIPd6; SlLIPe14; SlLIPa8; SlLIPb13; SlLIPa6; SlLIPe10; SlLIPe9;*	N/A	Stress Response
AT~TATA-Box	*SlLIPb32; SlLIPb31; SlLIPa26; SlLIPc22; SlLIPc21; SlLIPb30; SlLIPb29; SlLIPb28; SlLIPc20; SlLIPc19; SlLIPb27; SlLIPc18; SlLIPb26; SlLIPd18; SlLIPb25; SlLIPa24; SlLIPb24; SlLIPe31; SlLIPe31; SlLIPc17; SlLIPc16; SlLIPc15; SlLIPc14; SlLIPc12; SlLIPc11; SlLIPc10; SlLIPa23; SlLIPa22; SlLIPd17; SlLIPa21; SlLIPa20; SlLIPb23; SlLIPc8; SlLIPa19; SlLIPd15; SlLIPc7; SlLIPb22; SlLIPb21; SlLIPe29; SlLIPb20; SlLIPe28; SlLIPb19; SlLIPc6; SlLIPc6; SlLIPb18; SlLIPb17; SlLIPa17; SlLIPd13; SlLIPe26; SlLIPe25; SlLIPc5; SlLIPc3; SlLIPe27; SlLIPd13; SlLIPa16; SlLIPe24; SlLIPb16; SlLIPd12; SlLIPd11; SlLIPa15; SlLIPb15; SlLIPb13; SlLIPa14; SlLIPd10; SlLIPa12; SlLIPe23; SlLIPe22; SlLIPe21; SlLIPd9; SlLIPe20; SlLIPe19; SlLIPd8; SlLIPa11; SlLIPd7; SlLIPa10; SlLIPe17; SlLIPa9; SlLIPe16; SlLIPe15; SlLIPd6; SlLIPd6; SlLIPe14; SlLIPd5; SlLIPa8; SlLIPb12; SlLIPb13; SlLIPb10; SlLIPa7; SlLIPa6; SlLIPd4; SlLIPd3; SlLIPe13; SlLIPe12; SlLIPe11; SlLIPe10; SlLIPe9; SlLIPe8; SlLIPa5; SlLIPa4; SlLIPb8; SlLIPb7; SlLIPb6; SlLIPe5; SlLIPe4; SlLIPe2; SlLIPe2; SlLIPa3; SlLIPa2; SlLIPa1; SlLIPb5; SlLIPe1; SlLIPb4; SlLIPb3; SlLIPb1; SlLIPc22; SlLIPc1; SlLIPd1;*	N/A	N/A
AAGAA-motif	*SlLIPb32; SlLIPb31;SlLIPa26; SlLIPc22; SlLIPb30; SlLIPb29; SlLIPc20; SlLIPb26; SlLIPb24; SlLIPc17; SlLIPc15; SlLIPc14; SlLIPc12; SlLIPc11; SlLIPc10; SlLIPa22; SlLIPa20; SlLIPe30; SlLIPc8; SlLIPa19; SlLIPc7; SlLIPe28; SlLIPb19; SlLIPa18; SlLIPc6; SlLIPb18; SlLIPd13; SlLIPc5; SlLIPc3; SlLIPe27; SlLIPa16; SlLIPe24; SlLIPd12; SlLIPa15; SlLIPb14; SlLIPb13; SlLIPa14; SlLIPa13; SlLIPd10; SlLIPe23; SlLIPe19; SlLIPe18; SlLIPd7; SlLIPa9; SlLIPd6; SlLIPd5; SlLIPa8; SlLIPb12; SlLIPb9; SlLIPa7; SlLIPd4; SlLIPd3; SlLIPe12; SlLIPe9; SlLIPa5; SlLIPa4; SlLIPb7; SlLIPe7; SlLIPe6; SlLIPe5; SlLIPa3; SlLIPa2; SlLIPb4; SlLIPb2; SlLIPc22;*	N/A	Plant growth and development
Gap-box	*SlLIPb30; SlLIPc11; SlLIPb14; SlLIPd6; SlLIPe2; SlLIPd1;*	N/A	N/A
MYC	*SlLIPb32; SlLIPb31; SlLIPa26; SlLIPc21; SlLIPb30; SlLIPb29; SlLIPa25; SlLIPc20; SlLIPc19; SlLIPb27; SlLIPc18; SlLIPb26; SlLIPd18; SlLIPb25; SlLIPb24; SlLIPe30; SlLIPe31; SlLIPc16; SlLIPc15; SlLIPc14; SlLIPc13; SlLIPc11; SlLIPc10; SlLIPa22; SlLIPd17; SlLIPa21; SlLIPa20; SlLIPc9; SlLIPe30; SlLIPc8; SlLIPa19; SlLIPd16; SlLIPd15; SlLIPc7; SlLIPb22; SlLIPe29; SlLIPb20; SlLIPb19; SlLIPc6; SlLIPb17; SlLIPa17; SlLIPc4; SlLIPc3; SlLIPd13; SlLIPa16; SlLIPb16; SlLIPd12; SlLIPa15; SlLIPb15; SlLIPb14; SlLIPb13; SlLIPa14; SlLIPd10; SlLIPa12; SlLIPe23; SlLIPe22; SlLIPe21; SlLIPd9; SlLIPe20; SlLIPe19; SlLIPe18; SlLIPd8; SlLIPa10; SlLIPa9; SlLIPe16; SlLIPe15; SlLIPd6; SlLIPd5; SlLIPb12; SlLIPb13; SlLIPb10; SlLIPb9; SlLIPa7; SlLIPa6; SlLIPd4; SlLIPd3; SlLIPe13; SlLIPe12; SlLIPe11; SlLIPe10; SlLIPe9; SlLIPe8; SlLIPa4; SlLIPb8; SlLIPb7; SlLIPb6; SlLIPe7; SlLIPe6; SlLIPe4; SlLIPa2; SlLIPa1; SlLIPe1; SlLIPb4; SlLIPb3; SlLIPd2; SlLIPb2; SlLIPb1; SlLIPc22; SlLIPc1; SlLIPd1;*	N/A	Hormone responsive
W-Box	*SlLIPb31; SlLIPc19; SlLIPb27; SlLIPb26; SlLIPd18; SlLIPc16; SlLIPa23; SlLIPa22; SlLIPa21; SlLIPa20; SlLIPe28; SlLIPe25; SlLIPe27; SlLIPb16; SlLIPd12; SlLIPa15; SlLIPd10; SlLIPe18; SlLIPa9; SlLIPe15; SlLIPd6; SlLIPd5; SlLIPb10; SlLIPa7; SlLIPd3; SlLIPe11; SlLIPe9; SlLIPa5; SlLIPe7; SlLIPe3; SlLIPa3; SlLIPa1; SlLIPb4; SlLIPb3; SlLIPc1; SlLIPd1;*	N/A	N/A
STRE	*SlLIPb32; SlLIPc22; SlLIPb30; SlLIPa25; SlLIPb28; SlLIPb26; SlLIPb25; SlLIPe31; SlLIPe31; SlLIPc15; SlLIPc13; SlLIPc12; SlLIPc11; SlLIPc10; SlLIPa22; SlLIPd17; SlLIPa21; SlLIPa20; SlLIPc9; SlLIPd16; SlLIPc7; SlLIPb21; SlLIPb20; SlLIPb19; SlLIPa18; SlLIPc6; SlLIPa17; SlLIPe25; SlLIPc3; SlLIPd13; SlLIPe24; SlLIPd11; SlLIPb13; SlLIPa14; SlLIPd10; SlLIPa12; SlLIPe22; SlLIPe21; SlLIPd9; SlLIPe17; SlLIPa9; SlLIPd5; SlLIPb12; SlLIPb9; SlLIPa7; SlLIPd4; SlLIPd3; SlLIPe13; SlLIPe12; SlLIPa5; SlLIPb7; SlLIPe6; SlLIPe3; SlLIPa3; SlLIPb5; SlLIPb2; SlLIPb1; SlLIPc22; SlLIPc1;*	N/A	Stress Response
MYB	*SlLIPb32; SlLIPb31; SlLIPa26; SlLIPc22; SlLIPc21; SlLIPb30; SlLIPa25; SlLIPb28; SlLIPc20; SlLIPb26; SlLIPd18; SlLIPb25; SlLIPb24; SlLIPe31; SlLIPe31; SlLIPc17; SlLIPc16; SlLIPc14; SlLIPc13; SlLIPc10; SlLIPa23; SlLIPa22; SlLIPd17; SlLIPa21; SlLIPa20; SlLIPb23; SlLIPc9; SlLIPa19; SlLIPd16; SlLIPd15; SlLIPc7; SlLIPb22; SlLIPb21; SlLIPe29; SlLIPb20; SlLIPe28; SlLIPb18; SlLIPb17; SlLIPa17; SlLIPe25;*	N/A	N/A

	SlLIPc4; SlLIPc3; SlLIPd13; SlLIPa16; SlLIPe24; SlLIPd12; SlLIPd11; SlLIPb15; SlLIPb14; SlLIPb13; SlLIPa14; SlLIPd10; SlLIPa12; SlLIPe22; SlLIPe21; SlLIPd9; SlLIPe20; SlLIPe19; SlLIPd8; SlLIPd7; SlLIPa9; SlLIPe16; SlLIPe15; SlLIPd6; SlLIPd5; SlLIPa8; SlLIPb12; SlLIPb13; SlLIPb10; SlLIPb9; SlLIPa7; SlLIPd4; SlLIPd3; SlLIPe13; SlLIPe12; SlLIPe11; SlLIPe10; SlLIPe9; SlLIPe8; SlLIPb8; SlLIPb7; SlLIPe7; SlLIPe6; SlLIPe4; SlLIPe3; SlLIPa3; SlLIPa2; SlLIPa1; SlLIPe1; SlLIPb3; SlLIPd2; SlLIPb2; SlLIPb1; SlLIPc22; SlLIPc1; SlLIPd1;		
AT-rich element	SlLIPc22; SlLIPb27; SlLIPb25; SlLIPa24; SlLIPe31; SlLIPc15; SlLIPc10; SlLIPe30; SlLIPe29; SlLIPc6; SlLIPa17; SlLIPc5; SlLIPc3; SlLIPa12; SlLIPe22; SlLIPe21; SlLIPe19; SlLIPe18; SlLIPd8; SlLIPa11; SlLIPe15; SlLIPb9; SlLIPa7; SlLIPe9; SlLIPe8; SlLIPa4; SlLIPb6; SlLIPe5; SlLIPe1; SlLIPb4;	binding site of AT-rich DNA binding protein (ATBP-1)	Plant growth and development
CAAT-box	SlLIPb32; SlLIPb31; SlLIPa26; SlLIPc22; SlLIPc21; SlLIPb30; SlLIPb29; SlLIPa25; SlLIPb28; SlLIPc20; SlLIPc19; SlLIPb27; SlLIPc18; SlLIPb26; SlLIPd18; SlLIPb25; SlLIPb24; SlLIPe31; SlLIPe31; SlLIPc17; SlLIPc16; SlLIPc15; SlLIPc14; SlLIPc13; SlLIPc12; SlLIPc11; SlLIPa23; SlLIPa22; SlLIPd17; SlLIPb23; SlLIPc9; SlLIPe30; SlLIPc8; SlLIPa19; SlLIPd16; SlLIPd15; SlLIPc7; SlLIPb22; SlLIPb21; SlLIPe29; SlLIPb20; SlLIPe28; SlLIPb19; SlLIPa18; SlLIPa18; SlLIPc6; SlLIPb18; SlLIPb17; SlLIPa17; SlLIPd13; SlLIPe26; SlLIPe25; SlLIPc5; SlLIPc4; SlLIPc3; SlLIPe27; SlLIPd13; SlLIPa16; SlLIPe24; SlLIPb16; SlLIPd12; SlLIPd11; SlLIPa15; SlLIPa15; SlLIPb15; SlLIPb14; SlLIPb13; SlLIPa14; SlLIPa13; SlLIPd10; SlLIPa12; SlLIPe23; SlLIPe22; SlLIPe21; SlLIPd9; SlLIPe20; SlLIPe19; SlLIPe18; SlLIPd8; SlLIPa11; SlLIPd7; SlLIPa10; SlLIPe17; SlLIPa9; SlLIPe16; SlLIPe15; SlLIPd6; SlLIPe14; SlLIPd5; SlLIPa8; SlLIPb12; SlLIPb13; SlLIPb9; SlLIPa7; SlLIPd4; SlLIPd3; SlLIPe13; SlLIPe12; SlLIPe11; SlLIPe10; SlLIPe9; SlLIPe8; SlLIPa5; SlLIPa4; SlLIPb8; SlLIPb7; SlLIPb6; SlLIPe7; SlLIPe5; SlLIPe4; SlLIPe3; SlLIPe2; SlLIPa3; SlLIPa2; SlLIPa1; SlLIPb5; SlLIPe1; SlLIPb4; SlLIPb3; SlLIPd2; SlLIPb2; SlLIPb1; SlLIPc22; SlLIPc1; SlLIPd1;	common cis-acting element in promoter and enhancer regions	N/A
TATA-box	SlLIPb32; SlLIPb31; SlLIPa26; SlLIPc22; SlLIPc21; SlLIPb30; SlLIPb29; SlLIPa25; SlLIPb28; SlLIPc20; SlLIPc19; SlLIPb27; SlLIPc18; SlLIPb26; SlLIPd18; SlLIPb25; SlLIPa24; SlLIPb24; SlLIPe31; SlLIPe31; SlLIPc17; SlLIPc16; SlLIPc15; SlLIPc14; SlLIPc13; SlLIPc12; SlLIPa23; SlLIPa22; SlLIPd17; SlLIPa21; SlLIPa20; SlLIPb23; SlLIPc9; SlLIPe30; SlLIPc8; SlLIPa19; SlLIPd16; SlLIPd15; SlLIPc7; SlLIPb22; SlLIPb21; SlLIPe29; SlLIPb20; SlLIPe28; SlLIPb19; SlLIPa18; SlLIPc6; SlLIPb18; SlLIPa17; SlLIPd13; SlLIPe26; SlLIPe25; SlLIPc5; SlLIPc3; SlLIPd13; SlLIPa16; SlLIPe24; SlLIPb16; SlLIPd12; SlLIPd11; SlLIPa15; SlLIPb15; SlLIPb14; SlLIPb13; SlLIPa14; SlLIPd10; SlLIPa12; SlLIPe23; SlLIPe22; SlLIPe21; SlLIPd9; SlLIPe20; SlLIPe19; SlLIPe18; SlLIPd8; SlLIPa11; SlLIPd7; SlLIPa10; SlLIPe17; SlLIPa9; SlLIPe16; SlLIPd6; SlLIPd5; SlLIPa8; SlLIPb13; SlLIPb10; SlLIPb9; SlLIPa6; SlLIPd4; SlLIPd3; SlLIPe13; SlLIPe12; SlLIPe11; SlLIPe10; SlLIPe9; SlLIPe8; SlLIPa5; SlLIPa4; SlLIPb8; SlLIPb7; SlLIPb6; SlLIPe7; SlLIPe6; SlLIPe5; SlLIPe4; SlLIPe3; SlLIPa2; SlLIPa1; SlLIPb5; SlLIPe1; SlLIPd2; SlLIPb2; SlLIPb1; SlLIPc22; SlLIPc1;	core promoter element around -30 of transcription start	Hormone responsive
F-Box	SlLIPb32;	N/A	N/A
Box III	SlLIPb17;	protein binding site	N/A
A-box	SlLIPc22; SlLIPc19; SlLIPa22; SlLIPb20; SlLIPa16; SlLIPa14; SlLIPe22; SlLIPa8; SlLIPe6;	cis-acting regulatory element	N/A
ACE	SlLIPb28; SlLIPe30; SlLIPb22; SlLIPb19; SlLIPb17; SlLIPa17; SlLIPb16; SlLIPa14; SlLIPe15; SlLIPd5; SlLIPb13; SlLIPe2; SlLIPa2;	cis-acting element involved in light responsiveness	Light responsive
Box 4	SlLIPb32; SlLIPb31; SlLIPa26; SlLIPc22; SlLIPc21; SlLIPb30; SlLIPb29; SlLIPa25; SlLIPb28; SlLIPc20; SlLIPb27; SlLIPc18; SlLIPb26; SlLIPd18; SlLIPb25; SlLIPa24; SlLIPb24; SlLIPe31; SlLIPe31; SlLIPc17; SlLIPc16; SlLIPc15; SlLIPc14; SlLIPc13; SlLIPc11; SlLIPc10; SlLIPa23; SlLIPa22; SlLIPd17; SlLIPa20; SlLIPb23; SlLIPc9; SlLIPe30; SlLIPc8; SlLIPd16; SlLIPd15; SlLIPc7; SlLIPb22; SlLIPb21; SlLIPe29; SlLIPb20; SlLIPe28; SlLIPa18; SlLIPc6; SlLIPb17; SlLIPa17; SlLIPd13; SlLIPe25; SlLIPc5; SlLIPc4; SlLIPc3; SlLIPe27; SlLIPd13; SlLIPa16; SlLIPe24; SlLIPb16; SlLIPd12; SlLIPd11; SlLIPa15; SlLIPb15; SlLIPb14; SlLIPb13; SlLIPd10; SlLIPa12; SlLIPe22; SlLIPe21; SlLIPd9; SlLIPe20; SlLIPe19; SlLIPd8; SlLIPa11; SlLIPd7; SlLIPa10; SlLIPe17; SlLIPa9; SlLIPe16; SlLIPe15; SlLIPd6; SlLIPe14; SlLIPd5; SlLIPa8; SlLIPb12; SlLIPb10; SlLIPb9; SlLIPd3; SlLIPe13; SlLIPe11; SlLIPe8; SlLIPa4; SlLIPb8; SlLIPb7; SlLIPb6; SlLIPe5; SlLIPe4; SlLIPe2; SlLIPa3; SlLIPa2; SlLIPa1; SlLIPb5; SlLIPe1; SlLIPb4; SlLIPb3; SlLIPd2; SlLIPb2; SlLIPb1; SlLIPc1; SlLIPd1;	part of a conserved DNA module involved in light responsiveness	
AE-box	SlLIPb31; SlLIPb30; SlLIPb27; SlLIPb26; SlLIPd18; SlLIPb24; SlLIPc17; SlLIPc14; SlLIPa19; SlLIPc4; SlLIPc3; SlLIPb16; SlLIPd12; SlLIPa13; SlLIPe23; SlLIPd8; SlLIPa9; SlLIPe15; SlLIPb12; SlLIPb9; SlLIPa7; SlLIPa6; SlLIPa5; SlLIPa4; SlLIPb7; SlLIPb2; SlLIPc22; SlLIPd1;	part of a module for light response	
G-Box	SlLIPb32; SlLIPb31; SlLIPa26; SlLIPc22; SlLIPc21; SlLIPb29; SlLIPa25; SlLIPb28; SlLIPc19; SlLIPb27; SlLIPc18; SlLIPb25; SlLIPa24; SlLIPc17; SlLIPc14; SlLIPc13; SlLIPc12; SlLIPc11; SlLIPa22; SlLIPd17; SlLIPa21; SlLIPc9; SlLIPe30; SlLIPa19;	is-acting regulatory element involved in light responsiveness	

	SlLIPc7; SlLIPb21; SlLIPe29; SlLIPb20; SlLIPe28; SlLIPa18; SlLIPc6; SlLIPb17; SlLIPa17; SlLIPc5; SlLIPd13; SlLIPa16; SlLIPe24; SlLIPb16; SlLIPd11; SlLIPb15; SlLIPa14; SlLIPd10; SlLIPa12; SlLIPe22; SlLIPe21; SlLIPd9; SlLIPe18; SlLIPd8; SlLIPe17; SlLIPa9; SlLIPe15; SlLIPd6; SlLIPe14; SlLIPd5; SlLIPb12; SlLIPb13; SlLIPa6; SlLIPd4; SlLIPd3; SlLIPe13; SlLIPe13; SlLIPe11; SlLIPe10; SlLIPe9; SlLIPa5; SlLIPa4; SlLIPb7; SlLIPe7; SlLIPe6; SlLIPe4; SlLIPe3; SlLIPe2; SlLIPe2; SlLIPa3; SlLIPa2; SlLIPb5; SlLIPb3; SlLIPd2; SlLIPb2; SlLIPc22;		
AAAC-motif	*SlLIPb25;*	light responsive element	
AT1-motif	*SlLIPb31; SlLIPc22; SlLIPa25; SlLIPb28; SlLIPc13; SlLIPe30; SlLIPd15; SlLIPb16; SlLIPa14; SlLIPa11; SlLIPa10; SlLIPe17; SlLIPd4; SlLIPd3; SlLIPe9; SlLIPa4; SlLIPe2; SlLIPa3;*	part of a light responsive module	
chs-CMA1a	*SlLIPb31; SlLIPa26; SlLIPc20; SlLIPa20; SlLIPc7; SlLIPb19; SlLIPa18; SlLIPb18; SlLIPb17; SlLIPa17; SlLIPe26; SlLIPe25; SlLIPc4; SlLIPd13; SlLIPb16; SlLIPa15; SlLIPb15; SlLIPe19; SlLIPd7; SlLIPe14; SlLIPb10; SlLIPb9; SlLIPb7; SlLIPe2; SlLIPa1;*	part of a light responsive element	
GATA-motif	*SlLIPb32; SlLIPc21; SlLIPb29; SlLIPb28; SlLIPc18; SlLIPb26; SlLIPc17; SlLIPc16; SlLIPc15; SlLIPa20; SlLIPb23; SlLIPc9; SlLIPe28; SlLIPb19; SlLIPa17; SlLIPd13; SlLIPe24; SlLIPa15; SlLIPb14; SlLIPb13; SlLIPa12; SlLIPe18; SlLIPb12; SlLIPb13; SlLIPb9; SlLIPa7; SlLIPe9; SlLIPa5; SlLIPe1;*	part of a light responsive element	
GT1-motif	*SlLIPa26; SlLIPb29; SlLIPb28; SlLIPa24; SlLIPe31; SlLIPe31; SlLIPc12; SlLIPa21; SlLIPa20; SlLIPc9; SlLIPe30; SlLIPa19; SlLIPd16; SlLIPe29; SlLIPb19; SlLIPe26; SlLIPc4; SlLIPa16; SlLIPd11; SlLIPa15; SlLIPb15; SlLIPb14; SlLIPb13; SlLIPa12; SlLIPe22; SlLIPe20; SlLIPe19; SlLIPd8; SlLIPd7; SlLIPa9; SlLIPe15; SlLIPd6; SlLIPd5; SlLIPb13; SlLIPb9; SlLIPe13; SlLIPe12; SlLIPe10; SlLIPe9; SlLIPe8; SlLIPb8; SlLIPb7; SlLIPe5; SlLIPe2; SlLIPb2; SlLIPc22;*	light responsive element	
GA-motif	*SlLIPb32; SlLIPb29; SlLIPc18; SlLIPa24; SlLIPa23; SlLIPc9; SlLIPd15; SlLIPe28; SlLIPc4; SlLIPe21; SlLIPa10; SlLIPe15; SlLIPd6; SlLIPe9; SlLIPe1;*	part of a light responsive element	
I-box	*SlLIPa25; SlLIPb26; SlLIPa24; SlLIPc9; SlLIPe30; SlLIPe26; SlLIPe24; SlLIPd11; SlLIPd9; SlLIPa11; SlLIPa7; SlLIPa5; SlLIPb7; SlLIPb5; SlLIPb1;*	part of a light responsive element	
TCT-motif	*SlLIPa25; SlLIPc19; SlLIPb27; SlLIPb24; SlLIPe31; SlLIPc17; SlLIPc14; SlLIPa21; SlLIPa20; SlLIPb23; SlLIPc9; SlLIPd16; SlLIPc7; SlLIPb22; SlLIPb21; SlLIPe29; SlLIPb20; SlLIPb19; SlLIPa17; SlLIPd13; SlLIPe26; SlLIPb16; SlLIPd12; SlLIPa15; SlLIPa14; SlLIPd10; SlLIPa12; SlLIPd9; SlLIPe20; SlLIPe18; SlLIPd8; SlLIPa9; SlLIPd6; SlLIPe14; SlLIPd5; SlLIPe11; SlLIPa5; SlLIPa4; SlLIPb7; SlLIPe1; SlLIPb4; SlLIPb2;*	part of a light responsive element	
CATT-motif	*SlLIPa24; SlLIPc10;*	part of a light responsive element	
MRE	*SlLIPa26; SlLIPc22; SlLIPb25; SlLIPe31; SlLIPe31; SlLIPb23; SlLIPc8; SlLIPe26; SlLIPc4; SlLIPc3; SlLIPa16; SlLIPd12; SlLIPa12; SlLIPe16; SlLIPd6; SlLIPe11; SlLIPe10; SlLIPe9; SlLIPb8; SlLIPe7; SlLIPe3; SlLIPa2; SlLIPb1;*	MYB binding site involved in light responsiveness	
3-AF1 binding site	*SlLIPa6; SlLIPa4;*	light responsive element	
TCCC-motif	*SlLIPa24; SlLIPa16; SlLIPa15; SlLIPb15; SlLIPa6; SlLIPd3; SlLIPe12;*	part of a light responsive element	
Box II	*SlLIPb25; SlLIPc13; SlLIPd11; SlLIPd6; SlLIPb8; SlLIPe7;*	part of a light responsive element	
Sp1	*SlLIPc15; SlLIPc12; SlLIPa17;*	light responsive element	
ATCT-motif	*SlLIPc21; SlLIPc19; SlLIPb25; SlLIPe31; SlLIPc15; SlLIPa19; SlLIPe28; SlLIPb18; SlLIPd13; SlLIPb7;*	part of a conserved DNA module involved in light responsiveness	
Circadian	*SlLIPe31; SlLIPc13; SlLIPa17; SlLIPa16; SlLIPa15; SlLIPb15; SlLIPe19; SlLIPb10; SlLIPb7; SlLIPb6; SlLIPb2; SlLIPb1; SlLIPc1;*	cis-acting regulatory element involved in circadian control	Plant growth and development
ERE	*SlLIPb32; SlLIPb31; SlLIPa26; SlLIPc22; SlLIPc21; SlLIPb30; SlLIPb29; SlLIPc20; SlLIPb27; SlLIPc18; SlLIPd18; SlLIPa24; SlLIPe31; SlLIPe31; SlLIPc17; SlLIPc16; SlLIPc14; SlLIPc13; SlLIPc12; SlLIPc11; SlLIPa20; SlLIPb23; SlLIPa19; SlLIPd15; SlLIPb21; SlLIPe29; SlLIPb20; SlLIPe28; SlLIPa18; SlLIPc6; SlLIPb17; SlLIPa17; SlLIPe26; SlLIPe25; SlLIPc5; SlLIPd13; SlLIPe24; SlLIPd12; SlLIPd11; SlLIPa15; SlLIPb13; SlLIPd10; SlLIPa12; SlLIPe23; SlLIPe22; SlLIPe21; SlLIPe20; SlLIPe19; SlLIPe18; SlLIPd8; SlLIPa11; SlLIPd7; SlLIPe17; SlLIPe16; SlLIPe15; SlLIPd6; SlLIPe14; SlLIPd5; SlLIPb13; SlLIPa7; SlLIPa6; SlLIPd4; SlLIPd3; SlLIPe12; SlLIPe10; SlLIPe9; SlLIPe8; SlLIPb8; SlLIPb6; SlLIPe7; SlLIPe3; SlLIPe2; SlLIPa2; SlLIPb5; SlLIPe1; SlLIPb4; SlLIPb3; SlLIPc22; SlLIPd1;*	ethylene-responsive element	Hormone responsive
TGA-element	*SlLIPc19; SlLIPe31; SlLIPc13; SlLIPc12; SlLIPd15; SlLIPb20; SlLIPe27; SlLIPb16; SlLIPb15; SlLIPe22; SlLIPa9; SlLIPb12; SlLIPa7; SlLIPd3; SlLIPe4; SlLIPa1; SlLIPc1;*	auxin-responsive element	

AuxRR-core	*SlLIPb32; SlLIPc15; SlLIPb12;*	cis-acting regulatory element involved in auxin responsiveness	
CGTCA-motif	*SlLIPa26; SlLIPc21; SlLIPc19; SlLIPb25; SlLIPb24; SlLIPc15; SlLIPc14; SlLIPc13; SlLIPa23; SlLIPd17; SlLIPa21; SlLIPc9; SlLIPd16; SlLIPb22; SlLIPc6; SlLIPd13; SlLIPe26; SlLIPc5; SlLIPc4; SlLIPc3; SlLIPe24; SlLIPd11; SlLIPd11; SlLIPb15; SlLIPe22; SlLIPe21; SlLIPe19; SlLIPd8; SlLIPe17; SlLIPa9; SlLIPe16; SlLIPd5; SlLIPa8; SlLIPb8; SlLIPe4; SlLIPe3; SlLIPa3; SlLIPa1; SlLIPe1; SlLIPb3;*	cis-acting regulatory element involved in the MeJA-responsiveness	
TGACG-motif	*SlLIPa26; SlLIPc21; SlLIPc19; SlLIPb25; SlLIPb24; SlLIPc15; SlLIPc14; SlLIPc13; SlLIPa23; SlLIPa22; SlLIPd17; SlLIPa21; SlLIPc9; SlLIPd16; SlLIPb22; SlLIPb20; SlLIPc6; SlLIPd13; SlLIPe26; SlLIPc4; SlLIPe27; SlLIPe24; SlLIPb15; SlLIPa14; SlLIPe21; SlLIPe19; SlLIPd8; SlLIPd7; SlLIPe16; SlLIPd5; SlLIPa8; SlLIPa7; SlLIPd3; SlLIPb8; SlLIPe3; SlLIPa3; SlLIPd2; SlLIPb1;*	cis-acting regulatory element involved in the MeJA-responsiveness	
ABRE	*SlLIPb32; SlLIPb31; SlLIPa26; SlLIPc22; SlLIPb29; SlLIPa25; SlLIPb28; SlLIPc19; SlLIPb27; SlLIPc18; SlLIPb25; SlLIPa24; SlLIPe31; SlLIPc14; SlLIPc13; SlLIPc12; SlLIPc11; SlLIPa22; SlLIPd17; SlLIPa21; SlLIPc9; SlLIPe30; SlLIPa19; SlLIPc7; SlLIPb21; SlLIPe29; SlLIPb20; SlLIPe28; SlLIPb19; SlLIPc6; SlLIPb17; SlLIPa17; SlLIPc5; SlLIPd13; SlLIPa16; SlLIPe24; SlLIPb16; SlLIPb15; SlLIPa14; SlLIPa13; SlLIPd10; SlLIPa12; SlLIPd9; SlLIPe18; SlLIPd8; SlLIPe17; SlLIPa9; SlLIPe15; SlLIPd6; SlLIPe14; SlLIPd5; SlLIPb12; SlLIPb13; SlLIPd4; SlLIPd3; SlLIPe13; SlLIPe11; SlLIPe10; SlLIPe9; SlLIPa5; SlLIPb7; SlLIPe7; SlLIPe6; SlLIPe4; SlLIPe3; SlLIPe2; SlLIPa2; SlLIPb3; SlLIPd2; SlLIPb2; SlLIPc22;*	cis-acting element involved in the abscisic acid responsiveness	
TCA-element	*SlLIPb32; SlLIPc22; SlLIPc21; SlLIPa25; SlLIPc18; SlLIPb25; SlLIPa24; SlLIPc15; SlLIPc13; SlLIPd17; SlLIPc9; SlLIPe30 SlLIPd16; SlLIPc7; SlLIPa17; SlLIPe27; SlLIPa16; SlLIPd12; SlLIPb14; SlLIPa11; SlLIPa10; SlLIPb9; SlLIPa6; SlLIPd4; SlLIPd3; SlLIPe12; SlLIPa4; SlLIPb7; SlLIPe7; SlLIPe4; SlLIPa2; SlLIPd1;*	cis-acting element involved in salicylic acid responsiveness	
GARE-motif	*SlLIPc21; SlLIPa25; SlLIPa23; SlLIPa23; SlLIPd17; SlLIPb23; SlLIPa19; SlLIPe29; SlLIPe21; SlLIPe16;*	gibberellin-responsive element	
P-box	*SlLIPc20; SlLIPc18; SlLIPd18; SlLIPa24; SlLIPc12; SlLIPc10; SlLIPc9; SlLIPd13; SlLIPb16; SlLIPb14; SlLIPe23; SlLIPd9; SlLIPa11; SlLIPe14; SlLIPa8; SlLIPe13; SlLIPe8; SlLIPe5; SlLIPd2; SlLIPc1;*	gibberellin-responsive element	
O^2-site	*SlLIPc18; SlLIPc16; SlLIPa23; SlLIPa22; SlLIPd17; SlLIPa21; SlLIPc5; SlLIPb15; SlLIPe20; SlLIPb10; SlLIPd3; SlLIPb7; SlLIPe7; SlLIPe6;*	cis-acting regulatory element involved in zein metabolism regulation	Plant growth and development
GCN4_motif	*SlLIPc21; SlLIPb30; SlLIPc10; SlLIPa22; SlLIPc9; SlLIPd8; SlLIPa11; SlLIPb9; SlLIPa7; SlLIPe13; SlLIPb8; SlLIPb7; SlLIPe2; SlLIPb2;*	cis-regulatory element involved in endosperm expression	Plant growth and development
CAT-box,	*SlLIPa23; SlLIPb23; SlLIPc3; SlLIPa16; SlLIPe24; SlLIPd7; SlLIPd4; SlLIPe10; SlLIPb7; SlLIPe4;*	cis-acting regulatory element related to meristem expression	Plant growth and development
CCGTCC-box	*SlLIPc22; SlLIPa22; SlLIPa16; SlLIPa14; SlLIPe22; SlLIPa8; SlLIPa6;*	cis-acting regulatory element related to meristem specific activation	N/A
HD-Zip 1, HD-Zip 2	*SlLIPb28; SlLIPc17; SlLIPd15; SlLIPe26; SlLIPb14;*	element involved in differentiation of the palisade mesophyll cells	N/A
CCAAT-box	*SlLIPa25; SlLIPc16; SlLIPa21; SlLIPa20; SlLIPb23; SlLIPb20; SlLIPc4; SlLIPe20; SlLIPd8; SlLIPd7; SlLIPd6; SlLIPa7; SlLIPb1;*	MYBHv1 binding site	Plant growth and development
MBSI	*SlLIPb19; SlLIPb13; SlLIPe15; SlLIPa2; SlLIPb3; SlLIPb1;*	MYB binding site involved in flavonoid biosynthetic genes regulation	Stress Response
MSA-like	*SlLIPc19;*	cis-acting element involved in cell cycle regulation	N/A
ARE	*SlLIPb31; SlLIPb30; SlLIPb29; SlLIPb28; SlLIPb26; SlLIPd18; SlLIPb25; SlLIPb24; SlLIPc13; SlLIPc12; SlLIPc11; SlLIPa23; SlLIPb23; SlLIPa19; SlLIPd15; SlLIPb22; SlLIPb21; SlLIPe29; SlLIPb20; SlLIPe28; SlLIPc6; SlLIPb17; SlLIPe26; SlLIPe25; SlLIPc5; SlLIPc4; SlLIPa16; SlLIPb16; SlLIPd12; SlLIPa15; SlLIPb14; SlLIPa14; SlLIPa13; SlLIPd10; SlLIPa12; SlLIPe22; SlLIPe21; SlLIPe19; SlLIPa11; SlLIPe16; SlLIPd5; SlLIPb12; SlLIPb10; SlLIPe11; SlLIPe8; SlLIPb6; SlLIPe7; SlLIPe6; SlLIPe3; SlLIPa3; SlLIPa2; SlLIPa1; SlLIPe1; SlLIPb3; SlLIPd2; SlLIPb1;*	cis-acting regulatory element essential for the anaerobic induction	Stress Response
GC-motif	*SlLIPc13; SlLIPd7; SlLIPe17; SlLIPe14; SlLIPd4; SlLIPe10; SlLIPe10; SlLIPe2;*	enhancer-like element involved in anoxic specific inducibility	
MBS	*SlLIPa26; SlLIPe31; SlLIPe31; SlLIPc14; SlLIPc13; SlLIPa23; SlLIPa22; SlLIPa21; SlLIPb23; SlLIPb23; SlLIPc9; SlLIPa19; SlLIPd16; SlLIPd15; SlLIPb21; SlLIPb17; SlLIPc4; SlLIPc3; SlLIPa15; SlLIPb15; SlLIPb13; SlLIPe22; SlLIPa9; SlLIPe16;*	MYB binding site involved in drought-inducibility	

	SlLIPb12; SlLIPb13; SlLIPa7; SlLIPa5; SlLIPe7; SlLIPe6; SlLIPe4; SlLIPe3; SlLIPa2; SlLIPd2; SlLIPd1;		
WUN-motif	*SlLIPb32; SlLIPa26; SlLIPb30; SlLIPb29; SlLIPc20; SlLIPc18; SlLIPb26; SlLIPb25; SlLIPa24; SlLIPc17; SlLIPc15; SlLIPe30 SlLIPb21; SlLIPe28; SlLIPa18; SlLIPc5; SlLIPc4; SlLIPe24; SlLIPb16; SlLIPd12; SlLIPa15; SlLIPb14; SlLIPa12; SlLIPe20; SlLIPe19; SlLIPd8; SlLIPa11; SlLIPd7; SlLIPe16; SlLIPd6; SlLIPe14; SlLIPb12; SlLIPa7; SlLIPe8; SlLIPa4; SlLIPb8; SlLIPb7; SlLIPe7; SlLIPe2; SlLIPa3; SlLIPa2; SlLIPb4; SlLIPb3;*	wound-responsive element	
LTR	*SlLIPa25; SlLIPb28; SlLIPc19; SlLIPc18; SlLIPb26; SlLIPc15; SlLIPc12; SlLIPc11; SlLIPc7; SlLIPb20; SlLIPb18; SlLIPc5; SlLIPa16; SlLIPa16; SlLIPb14; SlLIPe18; SlLIPe15; SlLIPb10; SlLIPd4; SlLIPe13; SlLIPe10; SlLIPb6; SlLIPe6; SlLIPd2; SlLIPc22; SlLIPd1;*	cis-acting element involved in low-temperature responsiveness	
TC-rich repeats	*SlLIPc20; SlLIPc19; SlLIPe31; SlLIPe31; SlLIPc17; SlLIPa22; SlLIPe30 SlLIPa19; SlLIPb22; SlLIPa17; SlLIPd11; SlLIPa15; SlLIPa14; SlLIPd8; SlLIPa11; SlLIPd6; SlLIPd5; SlLIPe11; SlLIPe2; SlLIPb5; SlLIPe1; SlLIPb1;*	cis-acting element involved in defense and stress responsiveness	

Table S4. List of primers used for qRT-PCR.

Gene locus	Gene names	Forward primer (5'-3')	Reverse primer (5'-3')	Function
Solyc02g068070	*SlTGLa6*	TGGAATTGGACATGGGATG	GCAAGGCTGCTGATTTTACC	qRT-PCR
Solyc02g094040	*SlTGLa9*	AACAGGCAGCCAGTTCTATTG	GAATGAAGATTGCGATGACG	qRT-PCR
Solyc03g119720	*SlTGLa13*	TTGGCACAGATCAAGAACACT	TGATGACCTTGTCCTTACGC	qRT-PCR
Solyc11g032200	*SlTGLa25*	TTATGGAATGTGGAAGAACCTC	GGATTTCAACCCCTTTAGTGTA	qRT-PCR
Solyc12g038160	*SlTGLa26*	TGGTTCCTTTTCCCCATTA	TACGCCTCGCCTTCTGTC	qRT-PCR
Solyc11g030950	*SlACTIN7*	GATGAAGGACCTCTACGGTAAC	AAGTGCGAGAGAATATGTGGTG	qRT-PCR
Solyc03g119720	*SlTGLa13*	ACACGGGGGACGAGCTCGGTACC	CTCACCATGGTGTCGACTCTAGATTTGCC	Sub-Cellular
		ATCCATTTTCTCTCTCTATCTCGCC	CCTCTTTTCTCCTGTTCTT	Localization
Solyc02g094040	*SlTGLa9*	ACACGGGGGACGAGCTCGGTACC	CTCACCATGGTGTCGACTCTAGATTTCTG	Sub-Cellular
		GACTTTCTCCATTCTCTTTCTCTCT	ATTCCTCAATGATTTCTGC	Localization
